# The Role of NF-κB Signaling in the Maintenance of Pluripotency of Human Induced Pluripotent Stem Cells

**DOI:** 10.1371/journal.pone.0056399

**Published:** 2013-02-20

**Authors:** Osamu Takase, Masahiro Yoshikawa, Mana Idei, Junichi Hirahashi, Toshiro Fujita, Tsuyoshi Takato, Takayuki Isagawa, Genta Nagae, Hirofumi Suemori, Hiroyuki Aburatani, Keiichi Hishikawa

**Affiliations:** 1 Department of Advanced Nephrology and Regenerative Medicine, Graduate School of Medicine, University of Tokyo, Tokyo, Japan; 2 Department of Nephrology and Endocrinology, Graduate School of Medicine, University of Tokyo, Tokyo, Japan; 3 Department of Oral and Maxillofacial Surgery, Graduate School of Medicine, University of Tokyo, Tokyo, Japan; 4 Genome Science Division, Research Center for Advanced Science and Technology, University of Tokyo, Tokyo, Japan; 5 Institute for Frontier Medical Science, Kyoto University, Kyoto, Japan; University of Pécs Medical School, Hungary

## Abstract

NF-κB signaling plays an essential role in maintaining the undifferentiated state of embryonic stem (ES) cells. However, opposing roles of NF-κB have been reported in mouse and human ES cells, and the role of NF-κB in human induced pluripotent stem (iPS) cells has not yet been clarified. Here, we report the role of NF-κB signaling in maintaining the undifferentiated state of human iPS cells. Compared with differentiated cells, undifferentiated human iPS cells showed an augmentation of NF-κB activity. During differentiation induced by the removal of feeder cells and FGF2, we observed a reduction in NF-κB activity, the expression of the undifferentiation markers Oct3/4 and Nanog, and the up-regulation of the differentiated markers WT-1 and Pax-2. The specific knockdown of NF-κB signaling using p65 siRNA also reduced the expression of Oct3/4 and Nanog and up-regulated WT-1 and Pax-2 but did not change the ES-like colony formation. Our results show that the augmentation of NF-κB signaling maintains the undifferentiated state of human iPS and suggest the importance of this signaling pathway in maintenance of human iPS cells.

## Introduction

Mouse and human embryonic stem (ES) cells have differences in morphology, doubling times, and the expression of differentiation markers [1]. Self-renewal and the undifferentiated state of mouse ES cells depend on the activation of Stat3 by leukemia inhibitory factor (LIF) and Bmp4 signaling [2]–[3]. On the other hand, human ES cells require FGF2 signaling or ERK activation and co-operation with Activin/Nodal signaling [4], but not LIF/Stat3 and Bmp4 signaling. In 2006, Yamanaka et al. established induced pluripotent stem (iPS) cells [5], [6]. Human iPS cells overcome two main problems of human ES cells: ethical issues related to derivation from human embryos, and the potential rejection of ES cell-derived cells by the immune system of the host. Consequently, human iPS cells are a promising resource in the field of regenerative medicine [5], [6]. Although the function of human iPS cells and human ES cells is thought to be similar, an understanding of the molecular mechanism by which pluripotency and the undifferentiated state is maintained in human iPS cells is important for the clinical application of these cells.

NF-κB is a multi-functional transcription factor involved in various biological processes including inflammation, apoptosis, and immune regulation. NF-κB and Rel proteins are expressed in mammals: p65 (RelA), p50 (NF-IκB1), p52 (NF-κB2), c-Rel (Rel), and RelB [7]. The activation of NF-κB is triggered by the release of p50/RelA from the cytoplasm into the nucleus as a result of the degradation of IkappaBalpha (IκBα). The degradation of IκBα is regulated by various pathways, such as the phosphorylation of MAPK and IκB kinase (IKK) α/β. However, the functional role of NF-κB in maintaining the undifferentiated state of ES cells is controversial. Kim et al. reported the up-regulation of NF-κB upon the differentiation of mouse ES cells [8], and Torres et al. reported that Nanog maintains the undifferentiated state of mouse ES cells by inhibiting NF-κB [9]. On the other hand, Armstrong et al. reported that specific inhibition of NF-κB reduces expression of undifferentiation markers such as Oct4, Nanog, SSEA-4 and induces differentiation of human ES cells [4]. These reports suggest that NF-κB has different roles in mouse and human ES cells, but its role in human iPS cells remains to be clarified.

In the present study, we used monolayer differentiation and p65 siRNA, and compared the roles of NF-κB signaling and undifferentiated markers such as Nanog and Oct4, with undifferentiated human iPS cells. Our results indicate that NF-κB, especially p65, plays crucial role for maintenance of pluripotency of human iPS cells.

## Materials and Methods

### Reagents

We performed SSEA-4, TRA-1-60, and TRA-1-81 staining using the ES Cell Characterization Kit (Chemicon). For immunofluorescent Oct3/4 staining, Oct 3/4 (C-10): sc-5279 was purchased from Santa Cruz. For the small interfering (si) RNA studies, designed and validated siRNA specific for NF-κB p65 (sc-29410), and control non-targeting siRNA (sc-37007) were purchased from Santa Cruz [10].

### Cell Culture

Human iPS cells (253G1, 201B6, 201B7) [6] [11]–[12] were generously provided by Prof. Yamanaka (University of Kyoto). Human ES cells (KhES-1, 2 and 3) [13]–[14] were provided by Institute for Frontier Medical Science, Kyoto University. The use of human ES cell lines was conducted in accordance with ‘The Guidelines for Derivation and Utilization of Human Embryonic Stem Cells (2001)’ of the Ministry of Education, Culture, Sports, Science and Technology, Japan, after approval of The University of Tokyo/Office for Life Science Research Ethics and Safety. The iPS cells were established by the retroviral transduction of four transcription factors to human fibroblasts: Oct3/4, Sox2, Klf4, and c-Myc [5], [6], [15]. The iPS cells were cultured according to a previously reported protocol [16]. Briefly, feeder cells (SNL) were grown to sub-confluence in Dulbecco’s modified Eagle’s medium (DMEM) (GIBCO BRL, Grand Island, NY, USA) supplemented with 10% fetal bovine serum (FBS), 0.1 mM MEM non-essential amino acids (SIGMA), 2 mM L-glutamine, 100 U/mL penicillin, and 100 µg/mL streptomycin (GIBCO BRL) at 37°C in a 5% CO_2_ environment. The SNL feeder cells were mitotically inactivated prior to the iPS culture. The SNL cells were added to 10 µg/mL of mitomycin C (SIGMA), incubated for 2 hours, and then washed with PBS to remove the mitomycin C. For the passaging of the iPS cells, the cells were seeded on SNL feeder cells in gelatin-coated tissue culture dishes and grown in ES cell medium (ReproCELL, Yokohama, Japan) supplemented with 5 ng/mL recombinant FGF2 (ReproCELL) at 37°C in a 5% CO_2_ environment [6], [17], [18]. The medium was changed every other day. For monolayer differentiation, the harvested iPS cell colonies were seeded onto a dish without feeder cells and grown in DMEM supplemented with 10% FBS without FGF2 for 7 days.

### RNA Extraction and Oligonucleotide Microarray Analysis

The total RNA was extracted from iPS and ES cells using TRIZOL (Invitrogen, Carlsbad, CA, http://www.invitrogen.com) according to the manufacturer’s protocol. The purity of the RNA was checked using an Agilent 2100 Bioanalyzer (Agilent Technologies, Palo Alto, CA). We used the Human Genome U133A Plus2.0 arrays (Affimetrix, Santa Clara, CA), which contain almost 54,000 probe sets representing more than 47,000 transcripts including 38,500 well-characterized human genes [19]. The preparation of the cRNA and the hybridization of the probe arrays were performed according to the manufacturer’s protocols.

### siRNA Treatment

Human iPS cells, which grew to form small colonies for about 24 hours after seeding, were treated with both p65 siRNA and control siRNA, as previously reported [20]. Briefly, each siRNA treatments were gently mixed with each concentration with siRNA Transfection medium and reagent solution at room temperature for 30 minutes. The complex solution was added into ES cell medium of each iPS cells in 6-well plate. In this study, the siRNA treatment was performed twice every 36 hours.

### Electrophoretic Mobility Shift Assay (EMSA)

Nuclear extracts were isolated and examined for NF-κB and activator protein 1 (AP-1) DNA-binding activity as described previously [20], [21]. The sequences of the consensus double-stranded oligonucleotides used to detect the DNA binding sites were 5′-AGTTGAGGGGACTTTCCCAGGC-3′ for NF-κB and 5′-CGCTTGATGACTCAGCCGGAA-3′ for AP-1 (Santa Cruz). Consensus oligonucleotides biotinylated with a 3′-end DNA labeling kit (Pierce, IL, USA) were incubated with 7 µg of nuclear extracts and reaction solutions for 30 minutes on ice. In the competition assays, a 20-fold excess of unlabeled NF-κB consensus oligonucleotides was added to labeled NF-κB consensus oligonucleotides, confirming the specificity of DNA–protein binding in every experiment. The binding reactions were separated on a 6% polyacrylamide gel and were subsequently transferred electrophoretically to a nylon membrane. Biotinylated DNA was detected using a streptavidin-horseradish peroxidase conjugate and a chemiluminescent substrate (Lightshift Chemiluminescent EMSA Kit, Pierce).

### Western Blot Analysis

Cell extracts from the iPS cells were collected and subjected to Western blotting using previously described methods [22]. Equal amounts of protein (30 µg) as determined using the BCA Protein Assay (Pierce) were subjected to sodium dodecyl sulfate-polyacrylamide gel electrophoresis (SDS-PAGE). Each antibody purchased from Santa Cruz Biotechnology was diluted 1∶2000–4000 for the Western blot analysis. Reactivity was detected using the ECL Advance Western Blotting Detection Kit (GE Healthcare, UK).

### Alkaline Phosphatase Staining

The pluripotent status of stem cells was detected using the ALP Staining Kit (System Biosciences, CA) [5], [6]. The human iPS cells cultured on feeder layer were washed and then fixed with fix solution for 5 minutes. The fixed cells were responded with the prepared ALP substrate solution for 15 minutes, protected from light. The reaction was stopped with PBS. The red stained cell colonies were observed using a light microscope.

### Immunohistochemical Analysis

The expressions of NF-κB p65 and Oct3/4 were detected using the ABC high-HRP Immunostaining Kit (TOYOBO, Osaka, Japan). Both antibodies were purchased from Santa Cruz Biotechnology and were diluted 1∶200–500 for the immunohistochemical analysis.

## Results

### Characterization of Human iPS Cells

**Figure 1:**
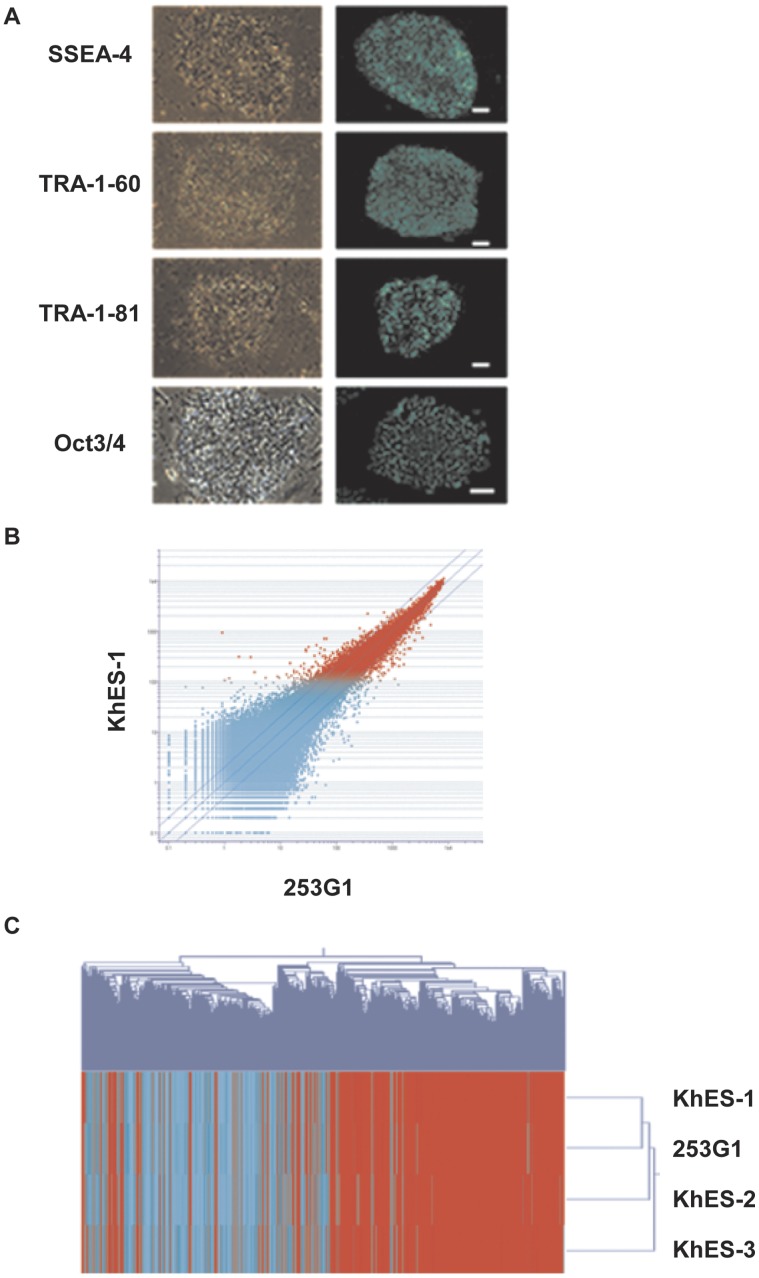
Characterization of human iPS cells. Representative phase contrast (left) and immunofluorescent (right) images of human iPS cells (253G1) grown on SNL feeder cells stained with SSEA-4, TRA-1-60, TRA-1-81, and Oct3/4. Scale bar, 100 µM (A). The global gene-expression patterns of human iPS cells (253G1) and human ES cells (KhES-1) were compared using oligonucleotide DNA microarrays (B). A Pearson correlation analysis was performed to cluster human iPS (253G1) cells and ES cells (KhES-1, 2 and 3). Red indicates increased expression, whereas blue means decreased expression (C).

Human iPS cells (253G1) were cultured on SNL feeder cells with ES cell medium containing FGF2. The undifferentiated state of the cell was confirmed using ES cell marker staining (SSEA-4, TRA1-60, TRA1-81, and Oct3/4) (Figure 1A). To confirm the character of the human iPS cells, we compared the global gene-expression profiles of human iPS cell and human ES cells (KhES-1, 2 and 3) (Figure 1B and C). A Pearson correlation analysis revealed that 253G1 was clustered closely with the human ES cells (Figure 1C). These results confirmed that the human iPS cells used in this study (253G1) were properly re-programmed and were similar to human ES cells.

### Down-regulation of NF-κB Activity and Undifferentiated Markers Upon Monolayer Differentiation

**Figure 2:**
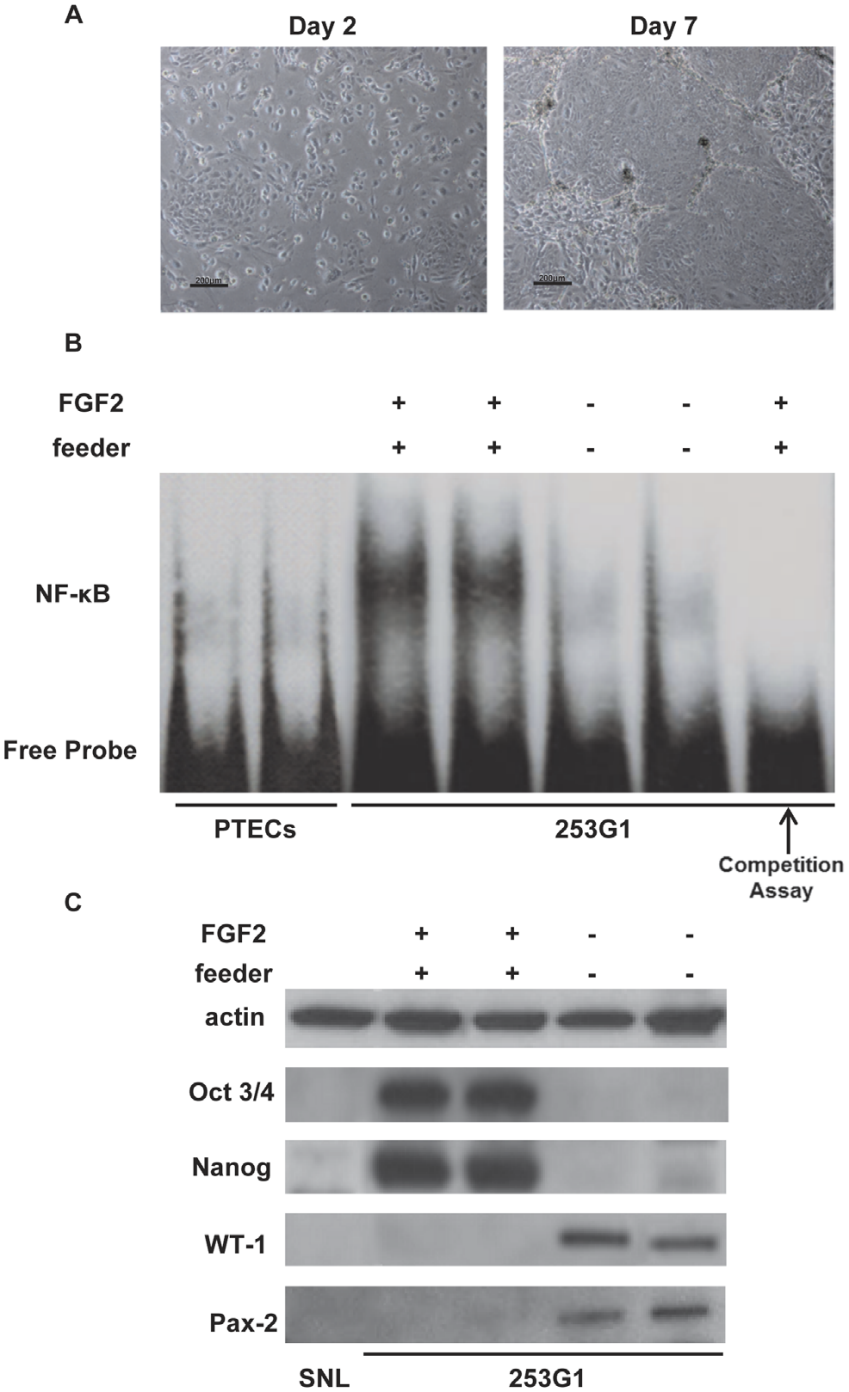
Down-regulation of NF-κB activity and undifferentiated markers upon monolayer differentiation. Representative photographs of monolayer differentiated cells (Days 2 and 7: original magnification, x200) (A). Representative electrophoretic mobility shift assays (EMSA) shows NF-κB binding activity in the undifferentiated iPS cells (FGF2+ and feeder+) and monolayer differentiated cells (FGF2- and feeder-). Proximal tubular epithelial cells (PTECs) were used as control cells (differentiated cells) for the EMSA. Results of 2 independent experiments are shown. In the last lane, the competition assays were performed using the undifferentiated iPS cells (B). Western blot analysis of actin, Oct3/4, NANOG, WT-1, and Pax-2 in the undifferentiated iPS cells (FGF2+ and feeder+) and monolayer differentiated cells (FGF2- and feeder-). Feeder cells (SNL) were used as negative control cells. Results of 2 independent experiments are shown (C).

For monolayer differentiation, human iPS cells were cultured without feeder cells using a standard culture medium (DMEM+10% FBS) without FGF2. Unlike the ES-like tightly packed flat colonies, the monolayer differentiated cells formed irregular colonies with large cells (Figure 2A). Compared with the differentiated cells (PTECs), NF-κB activity was augmented in the undifferentiated human iPS cells, but was almost abolished in the monolayer differentiated cells (Figure 2B). In the undifferentiated state, human iPS cells highly expressed Oct3/4, and Nanog, but did not express WT-1 or Pax-2. However, the monolayer differentiated cells highly expressed WT-1 and Pax-2, but did not express Oct3/4 or Nanog (Figure 2C).

### Specific Knockdown of NF-κB Activity by Treatment with p65 siRNA

**Figure 3:**
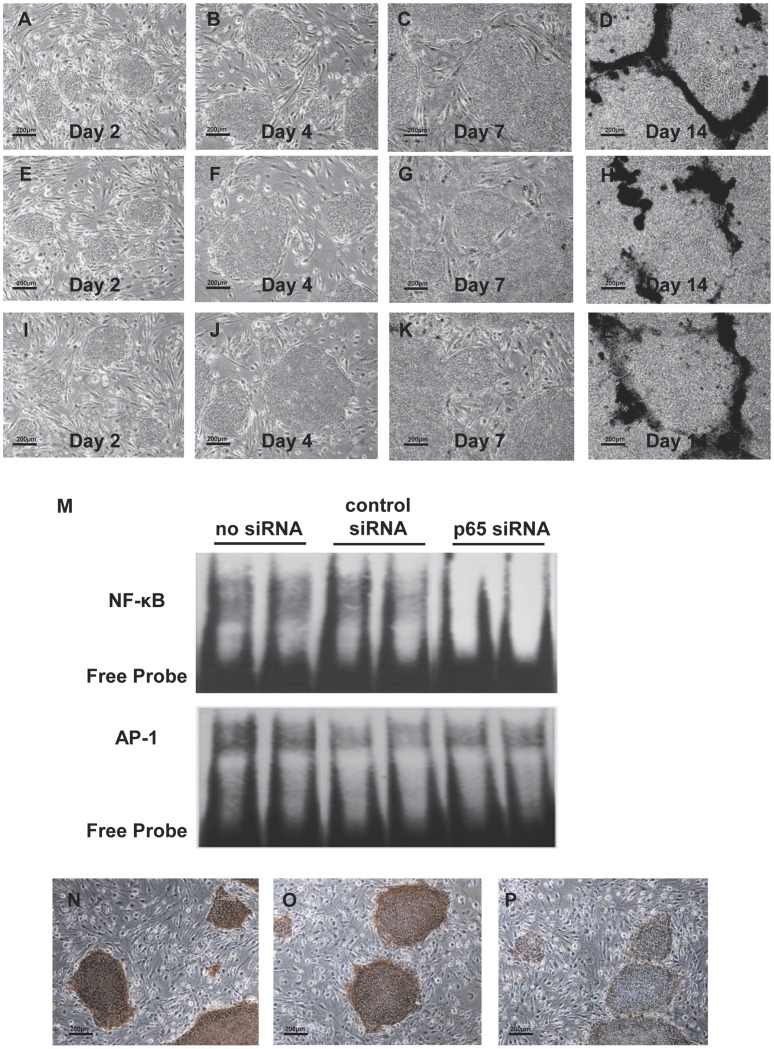
Specific knockdown of NF-κB activity by treatment with p65 siRNA. Representative photomicrographs of human iPS cells: no treatment (A–D), treatment with control siRNA (E–H), treatment wirth p65 siRNA (I–L) (original magnification, x200). A representative EMSA shows NF-κB and AP-1 binding activity in human iPS cells treated with siRNA (M). Representative photographs of alkaline phosphatase staining of human iPS cells treated with siRNA (N–P) (original magnification, x200). No treatment (N), treatment with control siRNA (O), treatment with p65 siRNA (P).

Human iPS cells cultured on SNL feeder cells were re-seeded on SNL feeder cells with ES cell medium in the absence or presence of p65 siRNA (Figure 3A–L). On day 4, treatment with p65 siRNA abolished the NF-κB activity but showed no effect on AP-1 binding as a control (Figure 3M). Unexpectedly, treatment with p65 siRNA showed no effect on cell growth and ES-like colony formation until day4 (Figure 3I and J). Compared with the no treatment and control siRNA treatments, the shape and size of the colonies were almost the same, but only the colonies treated with p65 siRNA were ALP negative (Figure 3N–P).

### Downregulation of Nanog and Oct3/4 by Knockdown of NF-κB

**Figure 4:**
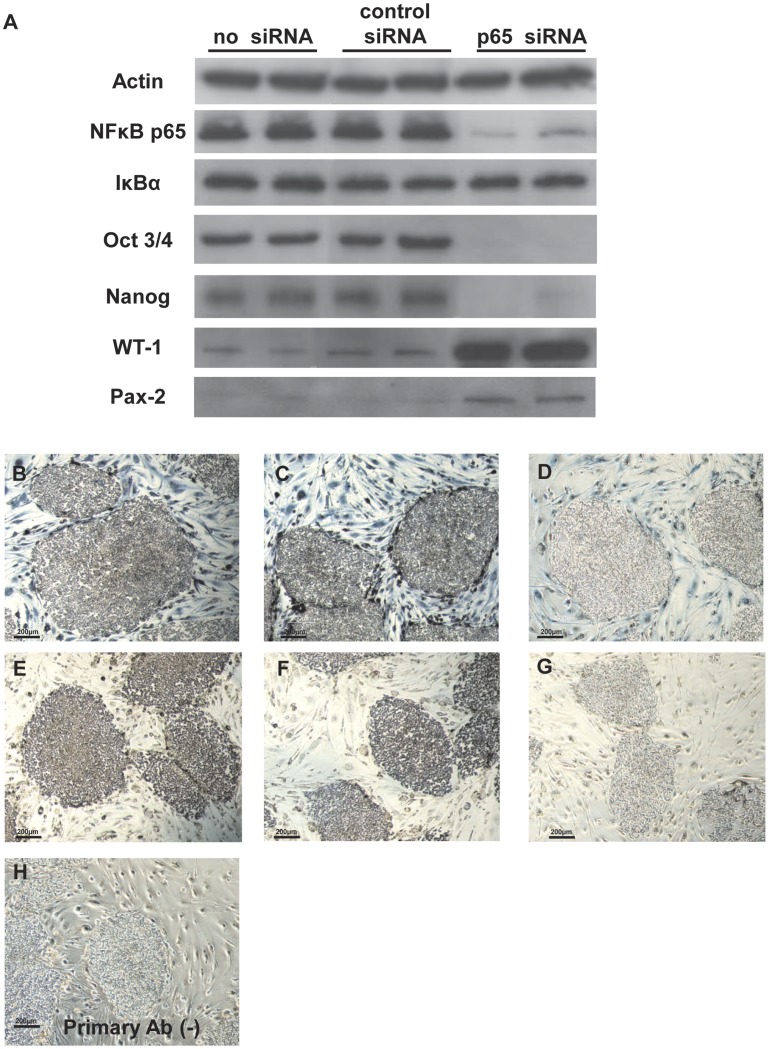
Downregulation of Oct3/4 and Nanog by knockdown of NF-κB. Western blot analysis of actin, NF-κB p65, IκBα, Oct3/4, Nanog, WT-1, and Pax-2 in human iPS cells treated with siRNA (A). Immunohistochemical staining for NF-κB p65 (B–D) and Oct3/4 (E–G) in human iPS cells treated with siRNA. No treatment (B and E), treatment with control siRNA (C and F), and treatment with p65 siRNA (D and G). Negative control staining without a primary antibody is shown (H) (Original magnification, x200).

To confirm the specific knockdown of p65 by siRNA, we performed a Western blot analysis. Treatment with p65 siRNA markedly reduced the protein expression of p65, but showed no effect on that of IκBα (Figure 4A). As in the case of monolayer differentiation, treatment with p65 siRNA abolished the expression of the undifferentiated markers Oct3/4 and Nanog and up-regulated those of the differentiation markers WT-1 and Pax-2 (Figure 4A). To clarify the down-regulation of p65 and Oct3/4 *in situ*, we performed immunohistochemistry studies. The no treatment colonies and the colonies that were treated with the control siRNA showed the strong expression of p65 and Oct3/4, but treatment with p65 siRNA markedly reduced the expression (Figure 4B–H).

## Discussion

Human iPS cells have great therapeutic potential, but the role of NF-κB signaling in the maintenance of their pluripotency has not been fully understood. NF-κB activity is reportedly inhibited in undifferentiated mouse ES cells, and NF-κB activity is repressed by Nanog [9]. On the other hand, in human ES cells, treatment with the specific NF-κB inhibitor, PDTC, reduces expression of undifferentiated markers such as Oct4, Nanog, SSEA-4 and promotes prominent differentiation [4]. Compared with human ES cells, we used properly reprogrammed human iPS cells (253G1) and demonstrated the augmentation of NF-κB activity in undifferentiated human iPS cells. The augmented NF-κB activity was also confirmed using another 3 independent human iPS cells lines (Figure S1). Furthermore, the knockdown of NF-κB down-regulated Nanog and Oct3/4 expression, suggesting that NF-κB activity in human iPS cells is not suppressed by Nanog. These results suggest that NF-κB signaling may play some different roles in the maintenance of an undifferentiated state in mouse ES cells and human ES cells, but we should think about epiblasts stem cell. As in the case with human ES and iPS cells, mouse epiblasts stem cells [23]–[24] rely on FGF signaling, form flat disc-shaped colonies *in vitro* and show poor clonal survival in the absence of the ROCK inhibitor Y-27632. Taken together, the likely explanation behind the different role of NF-κB is not explained by species differences but that mouse ES cells reside in a different pluripotent state relative to human ES and iPS cells.

Armstrong et al. reported specific inhibition of NF-κB induces prominent differentiation of human ES cells [4], but the same group also reported the activation of NF-κB signaling pathway during differentiation as a result of embryoid body (EB) formation [25]. As Armstrong et al. reported, we confirmed augmented NF-κB activity in undifferentiated human ES cells and down-regulation of NF-κB activity upon monolayer differentiation (Figure S1). We also confirmed augmented NF-κB activity in EB formation (Figure S1). This discrepancy may be explained by difference in culture condition. Inhibition of NF-κB using PDTC or siRNA was performed using 2-dimensional culture condition, but activation of NF-κB was confirmed using 3-dimensional culture condition, EB formation. As EB is a 3-dimensional mass, the culture condition of the cells inside of EB is not uniform. For example, oxygen concentration of center of EB is assumed to be much lower than outside of the mass. The cells outside of EB may utilize culture medium like monolayer 2-dimensional culture, but the cells located in the center of EB cannot directly contact or utilize culture medium. In other words, EB contains lots of cells cultured under different conditions and 2-dimensional monolayer culture condition may be more suitable to evaluate the role of NF-κB in the maintenance of pluripotency of human iPS cells.

Colony formation is one of the typical characteristics of undifferentiated ES and iPS cells, and an ES-like colony shape is the most potent indicators to select undifferentiated colonies during long-term ES cultures or the establishment of iPS cells. Interestingly, the knockdown of NF-κB by p65 siRNA abolished the expression of Oct3/4 and Nanog but never affected the colony growth and shape of human iPS cells until day 4. Since knockdown treatment with siRNA was transient (Figure S2) and the precise mechanism remains to be determined, but our results show a minimal role of Oct3/4 and Nanog in ES-like colony formation of human iPS cells.

WT-1 exhibits a spatially and temporally defined expression in the developing genitourinary system, and the WT-1 promotor contains a conserved NF-κB site [26]. The Pax-2 gene is a kidney-specific master gene that is required in both UB and mesenchymal cell lineages, normally optimizing UB branching and the mesenchymal-to-epithelial transformation in kidney development; several NF-κB binding motifs are present in the full length of the 5′-promotor region of Pax-2. However, our results showed that the specific knockdown of NF-κB up-regulated WT-1 and Pax-2 expression. Recently, Chen et al. reported that NF-κB is not required for regulation of expression of the WT-1 gene [26], and Pax-2 gene expression requires both ROS generation and NF-κB activation. Taken together, the expression of WT-1 and Pax-2 in human iPS cells may not be directly regulated by NF-κB.

In conclusion, our results demonstrate that NF-κB signaling plays key roles in maintaining the undifferentiated state of human iPS cells suggesting that this signaling pathway can be another important indicator of the maintenance of human iPS cells.

## Supporting Information

Figure S1 **The relation of NF-κB activity to differentiation and undifferentiation in various iPS and ES cells.** (A) Representative EMSA shows NF-κB binding activity in the undifferentiated iPS cells (Day 0: FGF2+ and feeder+) and monolayer differentiated cells (Day 7: FGF2- and feeder-). Results of four different kinds of human iPS cells (253G1, 201B6, 201B7 and R-iPS) are shown. EB means EB formation for 7 days. R-iPS was established from human renal epithelial cells (RPTECs). RPTECs and culture medium (REGM BulletKit) containing 0.5% FBS, hydrocortisone (0.5 mg/mL), epidermal growth factor (10 ng/mL), epinephrine (0.5 mg/mL), triiodothyronine (6.5 ng/mL), transferrin (10 mg/mL), insulin (5 mg/mL), gentamicin (50 mg/mL), and amphotericin (50 ng/mL) were purchased from Cambrex Corporation (East Rutherford, NJ). R-iPS cells were established by the retroviral transduction of four transcription factors: Oct3/4, Sox2, Klf4, and c-Myc. We confirmed pluripotency of R-iPS by expression of undifferentiated markers and teratoma formation. (B) Representative EMSA shows NF-κB binding activity in the undifferentiated human ES cells (Day 0: FGF2+ and feeder+) and monolayer differentiated cells (Day 7: FGF2- and feeder-). (C) Western blot analysis of actin, Oct3/4, NANOG, WT-1, and Pax-2 in the undifferentiated cells (Day 0: FGF2+ and feeder+) and monolayer differentiated cells (Day 7: FGF2- and feeder-). Results of four different kinds of human iPS cells (253G1, 201B6, 201B7 and R-iPS) and human ES cells are shown. (D-H) Whole blot panels of (C) are shown.
(TIF)

Figure S2 **Long time course of NF-κB activity with p65 siRNA.** A representative EMSA shows time course of NF-κB binding activity (Day 14 and Day 28) in human iPS cells (253G1) treated without (−) and with (+) p65 siRNA.
(TIF)

Figure S3 **Macro-scopic photograph of ALP staining and immunohistochemical staining.** Images of entire plates of ALP staining (A) and immunohistochemical staining for NF-κB p65 (B) and Oct3/4 (C) in human iPS cells (253G1) treated with siRNA.
(TIF)
