# Supplementary figures and images for: The Role of NF-κB Signaling in the Maintenance of Pluripotency of Human Induced Pluripotent Stem Cells
Source: PLoS One. 2013 Feb 20;8(2):e56399. doi: 10.1371/journal.pone.0056399 (PMC3577895; doi:10.1371/journal.pone.0056399)

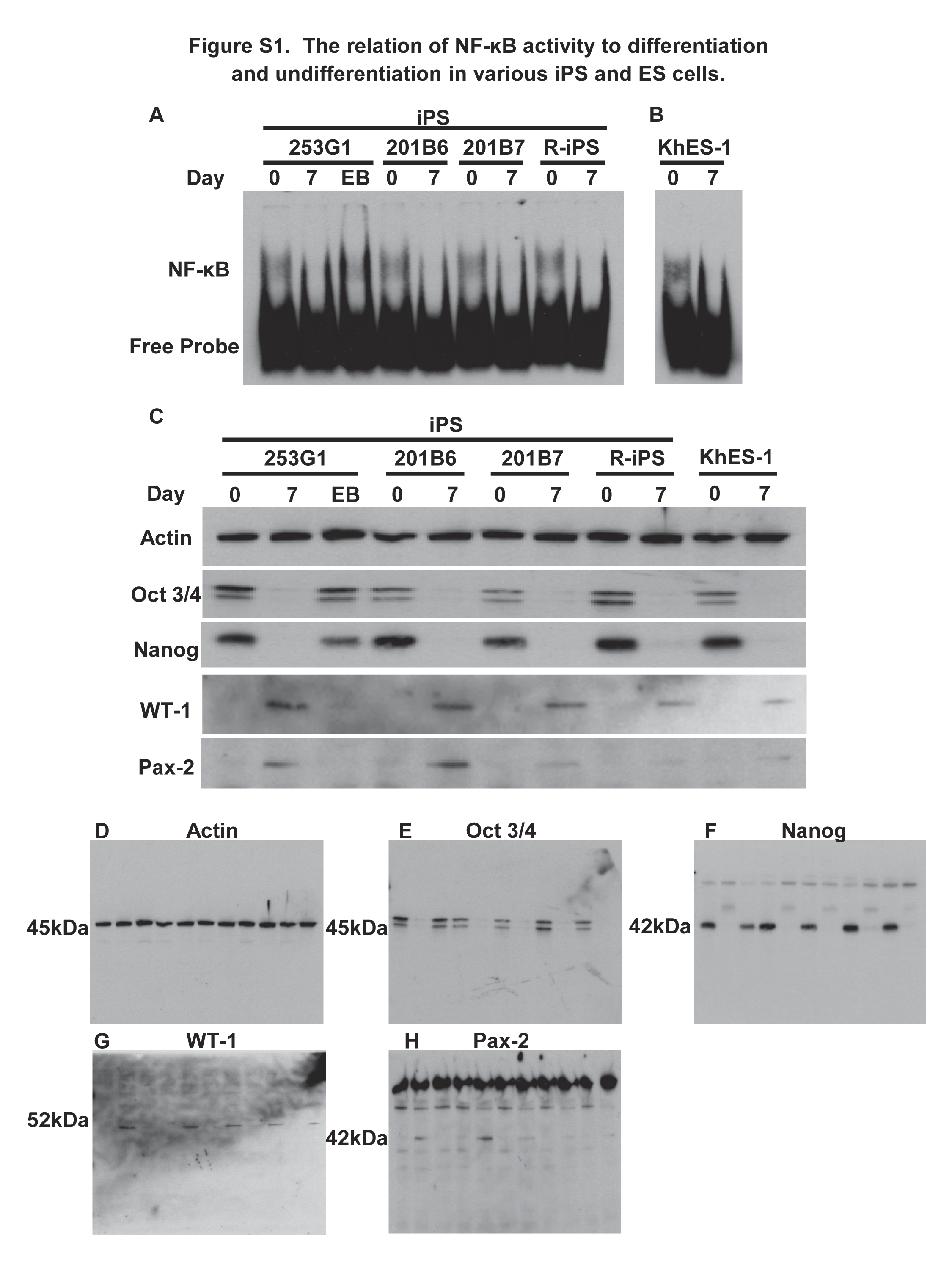

Supplement: Figure S1 — The relation of NF-κB activity to differentiation and undifferentiation in various iPS and ES cells. (A) Representative EMSA shows NF-κB binding activity in the undifferentiated iPS cells (Day 0: FGF2+ and feeder+) and monolayer differentiated cells (Day 7: FGF2- and feeder-). Results of four different kinds of human iPS cells (253G1, 201B6, 201B7 and R-iPS) are shown. EB means EB formation for 7 days. R-iPS was established from human renal epithelial cells (RPTECs). RPTECs and culture medium (REGM BulletKit) containing 0.5% FBS, hydrocortisone (0.5 mg/mL), epidermal growth factor (10 ng/mL), epinephrine (0.5 mg/mL), triiodothyronine (6.5 ng/mL), transferrin (10 mg/mL), insulin (5 mg/mL), gentamicin (50 mg/mL), and amphotericin (50 ng/mL) were purchased from Cambrex Corporation (East Rutherford, NJ). R-iPS cells were established by the retroviral transduction of four transcription factors: Oct3/4, Sox2, Klf4, and c-Myc. We confirmed pluripotency of R-iPS by expression of undifferentiated markers and teratoma formation. (B) Representative EMSA shows NF-κB binding activity in the undifferentiated human ES cells (Day 0: FGF2+ and feeder+) and monolayer differentiated cells (Day 7: FGF2- and feeder-). (C) Western blot analysis of actin, Oct3/4, NANOG, WT-1, and Pax-2 in the undifferentiated cells (Day 0: FGF2+ and feeder+) and monolayer differentiated cells (Day 7: FGF2- and feeder-). Results of four different kinds of human iPS cells (253G1, 201B6, 201B7 and R-iPS) and human ES cells are shown. (D-H) Whole blot panels of (C) are shown. (TIF) [file pone.0056399.s001.tif]

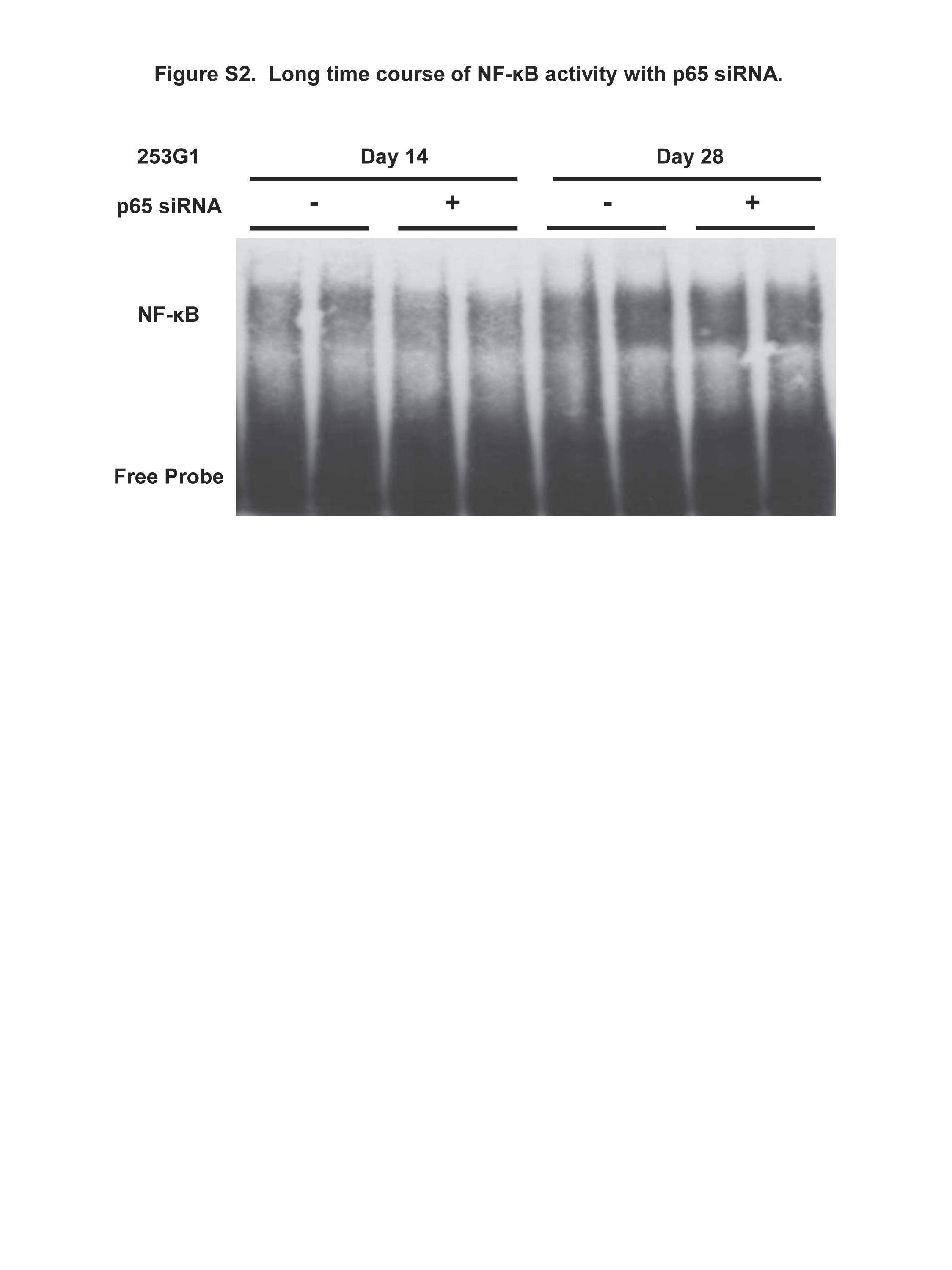

Supplement: Figure S2 — Long time course of NF-κB activity with p65 siRNA. A representative EMSA shows time course of NF-κB binding activity (Day 14 and Day 28) in human iPS cells (253G1) treated without (−) and with (+) p65 siRNA. (TIF) [file pone.0056399.s002.tif]

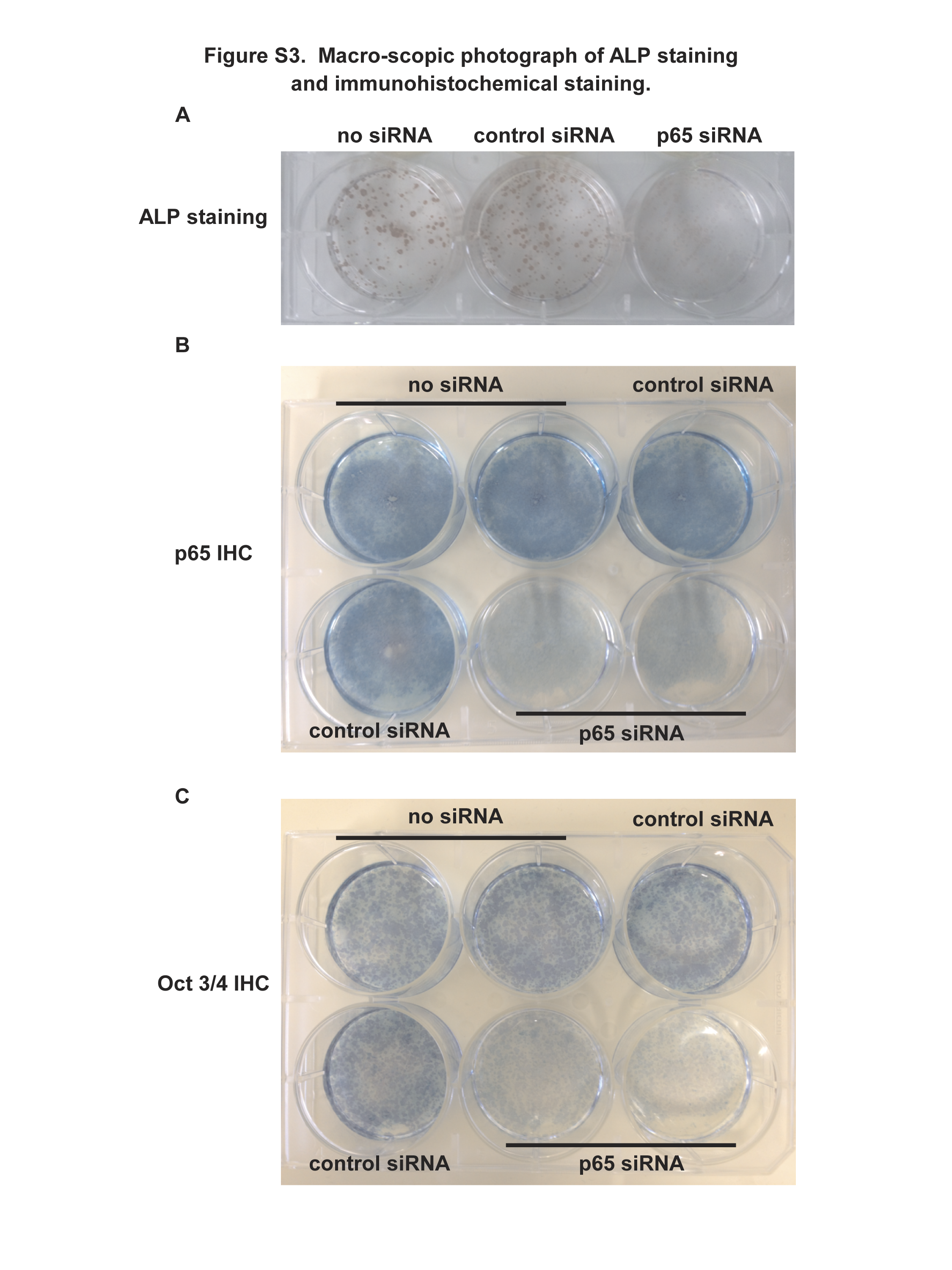

Supplement: Figure S3 — Macro-scopic photograph of ALP staining and immunohistochemical staining. Images of entire plates of ALP staining (A) and immunohistochemical staining for NF-κB p65 (B) and Oct3/4 (C) in human iPS cells (253G1) treated with siRNA. (TIF) [file pone.0056399.s003.tif]
